# Narratives of the Future Affect Fertility: Evidence from a Laboratory Experiment

**DOI:** 10.1007/s10680-021-09602-3

**Published:** 2022-02-07

**Authors:** Daniele Vignoli, Alessandra Minello, Giacomo Bazzani, Camilla Matera, Chiara Rapallini

**Affiliations:** 1 Department of Statistics, Computer Science, Applications “G. Parenti”, University of Florence, Viale GB Morgagni 59, 50134 Firenze, Italy; 2 Department of Statistical Sciences, University of Padova, Padua, Italy; 3 Department of Political and Social Sciences, University of Florence, Firenze, Italy; 4 Department of Education, Languages, Intercultures, Literatures and Psychology, University of Florence, Firenze, Italy; 5 Department of Economics and Management, University of Florence, Firenze, Italy

**Keywords:** Fertility intentions, Narratives of the future, Uncertainty, Expectations, Laboratory experimentation

## Abstract

In recent years, fertility rates have declined in most wealthy countries. This phenomenon has largely been explained by focusing on the rise of economic uncertainty. We contribute to this debate by arguing that, under uncertain conditions, *narratives of the future*—i.e., socially conveyed imagined futures—impact individuals’ decision-making about childbearing. To assess this impact, we conducted (for the first time in fertility intention research) a controlled laboratory experiment in two contrasting settings: Florence (Italy, *N* = 800) and Oslo (Norway, *N *= 874). Individuals were randomly exposed to a specific positive or negative future economic scenario (treatments) and were compared with individuals who were not exposed to any scenario (control group). Participants were then asked whether they intended to have a child in the next three years. The results showed a clear *causal* impact of narratives of the future on fertility intentions among the participants. Moreover, when the actual economic condition at the macro- (country context) or micro-level (labor-market status and characteristics) was more favorable, *negative* narratives of the future played a more crucial role. Conversely, when the actual economic conditions were less favorable, *positive* narratives of the future proved especially important. We conclude that, in the era of global uncertainty, individuals respond to more than their actual situation and constraints; narratives of the future create a *distance experience* from the daily routine that plays a potent role by inhibiting or facilitating fertility decision-making.

## Introduction

Since the late 1980s, a series of global transformations and structural shifts (i.e., the declining importance of national borders for economic transactions; the intensification of worldwide social relations through the information and technology revolution; the deregulation, privatization, and liberalization of national industries and markets; and the rising exposure to volatile job markets) have completely re-shaped domestic institutions beyond recognition (e.g., welfare regimes, employment, education, and transnational production systems) (Harvey, 2007). These globalization trends promised more competitive prices, wider choices, greater freedom, higher living standards, and increased prosperity. Indeed, wealthy societies have experienced advances in information and communication technologies, significant decreases in transportation costs, and increased purchasing power (Hartmann, 2014). Working conditions also improved during this period, with more regulation in work contracts, the reduction in working hours, longer periods of paid leave, and worker protection in the event of illness or maternity (Scherer, 2009). Nonetheless, globalization trends have also exacerbated the sources of uncertainty (Zinn, 2008) and have been accompanied by negative adjustments such as salary cuts, job losses, layoffs, bankruptcies, and business failures (Sennet, 1998; Bandelj et al., 2011; Mills & Blossfeld, 2013). Furthermore, the COVID-19 pandemic may have radically changed the European economic scenario for the following years due to its operating as a multiplier of uncertainty (Gieseck & Rujin, 2020; Luppi et al., 2021). Embedded in this contemporary scenario, fertility decisions are thus taken in a condition of rising uncertainty: as the future is less predictable, decisions are less based on individuals’ forecasting capacity.

Data trends have illustrated the decline, or stabilization, of total fertility in most European countries during the Great Recession and its aftermath (Comolli et al., 2021; Matysiak et al., 2021), for which economic uncertainty has been proposed as a central explanation (Kreyenfeld et al., 2012; Vignoli, Bazzani, et al., 2020). Family demographers have so far primarily operationalized the forces of economic uncertainty through objective indicators of individuals’ labor-market situations, such as temporary contracts or unemployment (Busetta et al., 2019; Kravdal, 2002; Kreyenfeld, 2010, 2015; Kreyenfeld et al., 2012; Mills & Blossfeld, 2013; Raymo & Shibata, 2017; Vignoli et al., 2012). However, while certainly not negligible, their negative impact on fertility has been proven to not be crucial (Alderotti et al., 2021). These studies have tended to view fertility decisions as an outcome of the “shadow of the past” (Davidson, 2010: p. 17; Beckert & Bronk, 2018), which is to say the result of previous events in a person’s life course (Johnson-Hanks et al., 2011). However, a fertility decision is, by its very nature, forward-looking. Consequently, the role of the “shadow of the future” (Huinink & Kohli, 2014: p. 1303; Bernardi et al., 2019: p. 4) cannot be ignored or downplayed. In reviewing the effects of recessions on fertility, Sobotka et al. (2011) emphasized the role of apprehension regarding future negative economic events in shaping fertility decisions. They suggested that individuals’ observations of the broader economic climate—including, crucially, media coverage—may serve to increase uncertainty and negatively affect fertility. Hence, individuals may be responding to more than their actual objective economic situation and economic constraints: *narratives of the future*—i.e., socially conveyed imagined futures shared by relevant others like parents and peers or the media—may play a larger role in people’s decision-making concerning childbearing (Vignoli, Bazzani, et al., 2020). Based on these shared narratives, individuals project themselves in an actionable imagined future (Beckert, 2016; Mische, 2009) and take decisions that may be more or less independent from their actual economic situation and structural constraints (i.e., employment or income).

This paper aims to promote the role of narratives of the future as a crucial lens with which to understand the connections between economic uncertainty and fertility intentions. Fertility intentions follow the desire for childbearing and anticipate concrete behavior by reflecting the combined effect of desired fertility and situational constraints (Billari et al., 2009; Thomson & Brandreth, 1995). To test the impact of the narratives of the future on fertility intentions, we conducted a controlled laboratory experiment in two “contrasting” settings: Italy and Norway. These two countries are extreme archetypical examples of social and family policies within the European context (Esping-Andersen, 1990, 1999; Javornik, 2014; Thévenon, 2011; United Nations, 2015). Nonetheless, they have been characterized by similar fertility declines since 2010; this relatively homogeneous fertility decline in both countries has been observed despite the persistence of a two-child family ideal (Sobotka & Beaujouan, 2014). Italy has been experiencing a constant fertility decline since 2010. The country re-entered the so-called lowest-low fertility regime in 2019, with a total fertility rate of 1.29. In Norway, the total fertility rate fell from a peak of almost two children per woman in 2009 to an all-time low of 1.53 in 2019. The fertility decline of Italy and Norway has been coupled with highly different economic trends. The Italian economy has experienced much turbulence since the 2008 Great Recession. In parallel, Norway, however, did not experience an economic recession—or, at least, not nearly to the same extent as the rest of Europe—and its GDP has increased every year since the Great Recession. Each study setting thus represents a unique pattern of pre-experimental conditions, influenced by a distinct set of cultural, political, and economic developments. In line with other comparative studies employing an experimental approach (D’Attoma et al., 2019; Pampel et al., 2019), our design intends to highlight the impact of narratives of the future on fertility intentions in two different settings.

The laboratory experiments were held in Florence and Oslo. Two samples included, respectively, 800 participants (both members of 400 heterosexual couples) in Florence, and 874 participants (both members of 437 heterosexual couples) in Oslo, with different labor-market conditions (jobless, employed with a time-limited contract, and employed with a permanent contract). Despite the limited external validity of our experimental design, this approach has clear advantages. When used with participants who vary in theoretically relevant ways, this type of approach allows researchers to both investigate causal relations, and assess the potential interactions between experimental conditions and both individual and contextual factors (Jackson & Cox, 2013). Two-thirds of our participants were randomly exposed to a narrative of the future in the form of a mock newspaper article describing a positive or negative economic scenario. The remaining third (serving as a control group) was not exposed to any scenario. After reading the mock article, participants were asked to envisage themselves in the scenario described and state whether they intended to have a child in the next three years. These fertility intentions were compared to those of the control group. The results suggest a clear causal impact of economic narratives of the future on fertility intentions in both countries. Additionally, we found that, under more favorable actual economic conditions at the macro- (country context) or micro-level (labor-market status and characteristics), the *negative* narrative of the future is most crucial. When the actual economic conditions are less favorable, the *positive* narrative of the future proves especially important. In addition to the counterfactual approach of our experimental strategy, the validity of our findings was reinforced by controlling the estimates for several markers of individual traits, partners’ characteristics, and structural constraints usually employed in the literature (e.g., risk aversion, and labor and income conditions).

The remainder of the paper is structured as follows. Section 2 introduces the background literature and research questions. Section 3 presents the laboratory experiment and our analytical strategy. Section 4 illustrates our results, which are subsequently discussed in Sect. 5. Details about the experimental protocol are provided in the Appendix.

## Narratives of the Future

Prior empirical evidence suggests that one’s individual background—e.g., parity, relationship status, level of education (Mencarini et al., 2015; Dommermuth et al., 2011)—and personality traits—e.g., risk aversion (Bellani & Arpino, 2021)—impact fertility intentions and behavior. Further to these forces, the effects of economic uncertainty on fertility have previously been assessed through the lens of cumulative life course experiences (Busetta et al., 2019; Özcan et al., 2010), actual labor and economic conditions (Kreyenfeld et al., 2012), and their perceptions (e.g., Fahlén & Oláh, 2018; Modena et al., 2013). The salience of economic uncertainty, however, depends not only on the objective characteristics of the economic situation, but also on socially constructed future expectations. Economic uncertainty is an inherently forward-looking notion, which requires a framework that acknowledges its prospective nature.

The study of subjective perceptions of economic factors and their influence on behavior has been discussed in the literature. The Subjective Expected Utility (SEU) model, for example, focuses on the expected utility of individual behavior, with different levels of emphasis placed upon its bounded nature (Simon, 1964), and the role of frames and habits (Esser, 1993). Within the New Home Economics (NHE) (Becker, 1964: p. 1981), fertility decisions are considered to be rational evaluations of the future expected utility of having children, with people calculating the trade-off between working and having a child. Despite the existence of empirical evidence for this kind of substitution effect, the application of a strict economic approach to fertility behavior may create a stylized and unrealistic type of family agency, in which partners meticulously calculate the costs and benefits of having a child, while discounting the actual cost in light of future utility. Fertility decisions are complex decisions which involve the interaction of interests, values, opportunities, and social ties. Then, Huinink and Kohli (2014) have recognized the importance of the “shadow of the future” in fertility plans within a risk framework, which is less restrictive than the SEU models (Fischhoff et al., 1981). In line with the risk framework, they assume fertility plans to be the effect of individuals “striving for subjective wellbeing (welfare production) as efficiently as they are able to” and evaluating costs and benefits of their child “investment” (Huinink & Kohli, 2014: 1298), but they also recognized that “in many ways, the benefits (and costs) of fertility, as compared to other domains of welfare production, are incommensurable” (ibid: 1302). This engenders the “‘veil of undecidability’ that makes actors receptive to relevant events or influences (e.g., from close peers) that may push them from one side to the other of the decision” (ibid: 1304). Fertility decisions are thus not a rational calculus between costs and benefits, because they are always taken in conditions of uncertainty rather than risk (Vignoli, Bazzani, et al., 2020). The probability distribution of different outcomes can be estimated for decisions taken within conditions of risk, whereas decisions taken in conditions of uncertainty are characterized by unknown probability distributions of future outcomes and are guided by imagined futures that can be more or less plausible and normatively oriented (Beckert, 2016; Tuckett & Nikolic, 2017).

Uncertainty is a crucial element for such long-term decisions as childbearing, because it is both an intrinsic characteristic of the future and a contingent condition of the present. As to the former, uncertainty is the precondition for the decision process: when people perceive the future to be uncertain, what was expected as the outcome of the ordinary routine seems no longer applicable, and a new deliberation is necessary (Dewey, 1930; Mead, 1932).^1^ Uncertainty in fertility decisions encapsulates more than the future economic situation. Indeed, it may concern several prospects, such as health, partner relationship, household labor division, work-family conciliation, and so forth. Economic uncertainty as a contingent condition of the present, instead, has become more salient in the recent decades of globalization and neoliberal policies (Harvey, 2007; Zinn, 2008). The influence of uncertainty on fertility is far from deterministic, however. Given a specific set of opportunities and constraints, fertility choices may well be influenced by socially conveyed narratives of the future that potentially encourage or discourage childbearing intentions (Vignoli, Guetto, et al., 2020). These shared narratives of the future may produce real effects on individuals’ decision-making processes, irrespective of their level of truth, rationality, or plausibility (Beckert, 2016; Mische, 2009; Tavory & Eliasoph, 2013; Gatta et al., 2021).^2^

On the one hand, and in line with Mills and Blossfeld’s (2013) globalization perspective, negative narratives of the future could impact fertility decisions, as young adults are more likely to postpone partnership and parenthood commitments when facing growing economic and temporal uncertainty, as demonstrated by previous literature based on objective measures of uncertainty, such as youth unemployment, term-limited working contracts, and unstable employment situations (Adsera, 2004, 2011; Kreyenfeld & Andersson, 2014; Neels et al., 2013; Özcan et al., 2010; Pailhé & Solaz, 2012). The negative perception of their future development thus potentially discourages fertility. On the other hand, narratives of the future could be considered powerful anti-uncertainty devices (Boyer, 2018), favoring childbearing even in adverse conditions. According to the socio-psychological uncertainty reduction framework developed by Friedman et al. (1994), having children may serve to reduce biographical uncertainty. This framework contends that uncertainty reduction is a universal immanent value driving the choices of all rational actors, and “having a child changes life from uncertain to relatively certain” (Friedman et al., 1994: p. 383). It advances a possible interpretation of the fertility decisions of women with limited labor-market options (Kreyenfeld, 2010; McDonald, 2000), who may respond to unfavorable employment prospects by choosing the “alternative career” of motherhood so as to lend structure to an otherwise uncertain life course. There is empirical evidence with which to support such an argument: Edin and Kefalas (2005), for instance, showed that the poorest US women in non-permanent employment may decide to have a child before marriage, because motherhood may increase their social status and serve to secure their future. Kreyenfeld (2010) found that economic uncertainty facilitated childbearing among poorly educated women living in Germany.

Shared narratives of the future are popularized by relevant others, such as parents or peers. More recently, the diffusion of social media allows narratives to circulate socially (Johnson et al., 2020), providing unprecedented access to the opinions and experiences of relevant others. The different narratives conveyed by the media may play a central role in orienting the decision process through a “framing” effect of the expected situation (Entman, 1991, 1993; Goffman, 1974). For the majority of citizens, the media is the primary source of information regarding the economic sphere (Joris et al., 2018), and it thereby affects individuals’ opinions and attitudes (Joris et al., 2018; Robins & Mayer, 2000; Thibodeau & Boroditsky, 2011). Schneider (2015) suggested that press coverage of the economy can more accurately measure the sentiments that shape economic uncertainty and affect fertility behaviors than objective indicators such as unemployment and foreclosures. Comolli and Vignoli (2021) showed that the general public responds to the media’s framing of uncertainty, with implications for childbearing. Interestingly, the results obtained within communication research suggest that negative news has a stronger impact on perceptions than positive reports. For instance, asymmetric effects have been demonstrated on consumer confidence (Alsem et al., 2008) and inflation (Dräger, 2015). This asymmetry can be explained by the prospect theory (Kahneman & Tversky, 1979), which asserts that loss aversion causes bad news to have a stronger impact than good news.

**Fig. 1:**
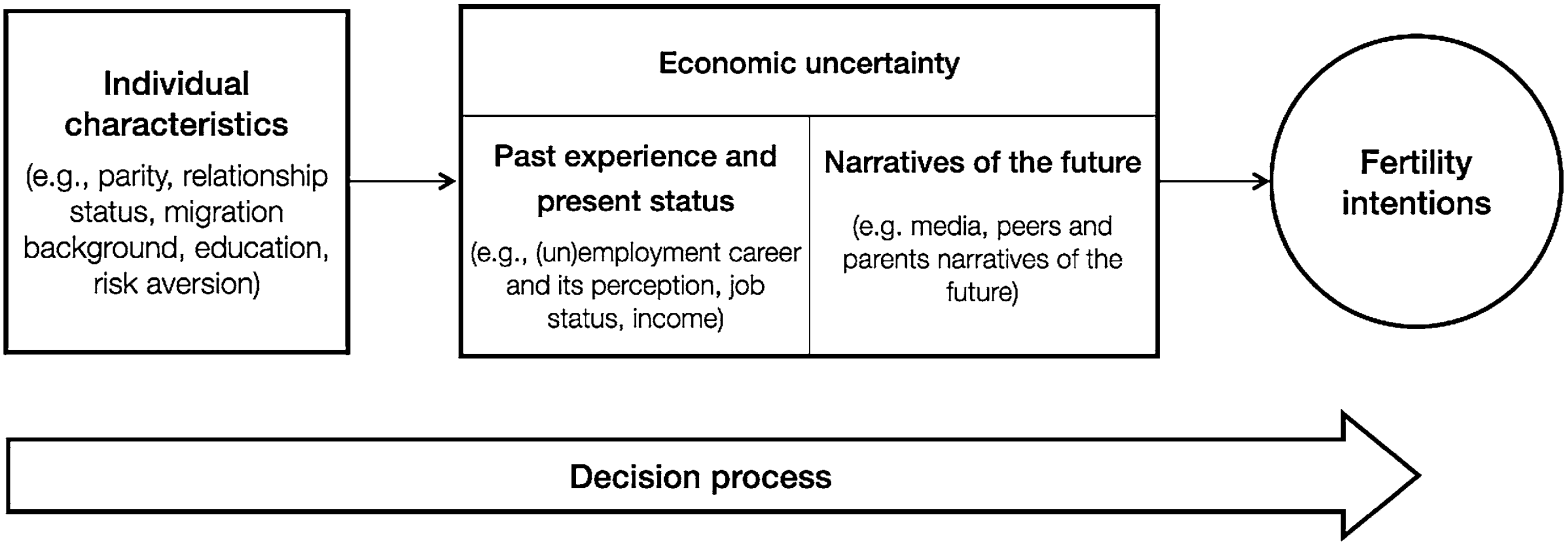
Stylized representation of the role of economic uncertainty in the fertility decision-making process

Figure 1 displays a stylized representation of the influence of economic uncertainty in the fertility-decision process. Past and current economic constraints, defined by objective indicators of the individual’s labor-market situation and perception, are coupled with shared narratives of the future conveyed by relevant others and the media. In what follows, we address a specific research question (RQ#1): *What is the causal effect of economic narratives of the future on individuals’ fertility intentions*? In line with the globalization perspective, we expect that a positive economic narrative of the future facilitates fertility intentions among both men and women (HP1a). Conversely, we expect that a negative economic narrative of the future inhibits fertility intentions among men (HP1b). Among women, such a negative narrative might have either a negative effect (HP1c) or—in line with the uncertainty reduction framework—a positive effect (HP1d).

## Moderators

Individuals’ judgment relies on available, accessible, and seemingly relevant information rather than on more abstract parameters. This relevant information refers primarily to familiar events and situations to which individuals are exposed, and are used as contrasts through which to evaluate the novelty’s salience and expected effect (Schwarz & Bless, 1992). In the decision-making process, different narratives of the future are different sources of “distance experience” from ordinary life (Dewey, 1930: p. 58; Mische, 2009: p. 697). Hence, notwithstanding the prominent role that narratives of the future may have in shaping the expected future (Beckert, 2016), the degree of the expected novelty conveyed by the narrative might exacerbate or mitigate its impact on individual fertility decision-making. Such a moderation effect may be observed both at the macro- and micro-levels.

### The studied settings

The Great Recession—apart from leading to a sharp increase in material deprivation and its subsequent downturn in both economic and labor-market trends—generated a narrative of the future characterized by rising uncertainty (Schneider & Hastings, 2015). Such a narrative may be perceived differently by individuals according to their country’s economic performance and level of economic resilience. We thus ask whether economic narratives of the future influence men’s and women’s fertility planning differently depending on their country of residence.

Comparative laboratory experimentation is a subject at the forefront of academic debate. We adopted a cross-country comparative design to account for the effect of real-world pre-treatment conditions, such as the differences in institutional settings and economic conditions (Weber & Hsee, 1998; Henrich et al., 2005; Norenzayan & Heine, 2005; Rieger et al., 2014; Zhang et al., 2014). We have focused on two contrasting settings—Florence and Oslo. The strength of the association between fertility intentions and subsequent realizations seems rather similar in Italy and Norway. Régnier-Loilier and Vignoli (2011) found that, in Italy, 62% of those that *definitely intended* to have a child within the next three years actually did so. Dommermuth et al. (2015) showed that 57% of those that *intended*^3^ to have a child in the next four years, did actually have one.

Both countries are well-known in the literature as being characterized by different welfare systems. Norway, classified by Esping-Andersen (1990, 1999) as a social-democratic welfare regime, has developed a comprehensive set of social services for working parents, thereby supporting their full-time integration into the labor-market (Thévenon, 2011). In contrast, low institutional support for working parents is characteristic of the familial welfare regime (such as Italy), where the provision of care from within the extended family is traditionally the norm (Esping-Andersen, 1990, 1999; Javornik, 2014). While Norwegian family policies are specifically designed to improve the reconciliation and balance of paid work, family life, and childcare choices for parents (Lappegård, 2010), Italy lacks a coherent system of policies with which to support childbearing (United Nations, 2015). Aside from these ample welfare differences, both countries have also shown resilience in the face of economic downturns, though to very different extents. Italy is one of the European countries that suffered the most from the consequences of the Great Recession (Coletto, 2020). Youth unemployment (individuals aged between 15 and 24) increased by over 15% between 2008 and 2014, when it peaked at 42.7% (Bank of Italy, 2020). Located at the other extreme of Europe, Norway suffered a very mild recession (Bell & Blanchflower, 2010). The Norwegian youth unemployment rate dropped at the beginning of the 2000s and reached its lowest level (7.37%) in 2007, rising to a high of 11.06% in 2016 (Plecher, 2020).^4^

Each country’s economic situation may contribute to framing the salience of the news. Our second research question is thus (RQ#2): *Does the country-context moderate the impact of (positive/negative) narratives of the future on fertility intentions?* There are two possible directions for such a moderation effect. First, a positive or negative economic narratives of the future might *amplify* the positive or negative role of the macro-economic context of the country on fertility intentions (HP2a). Second, the effect of the narrative of the future could generate a contrast principle (Cialdini & Cialdini, 2007): Where the economic trend is turbulent, a positive economic narrative can be a source of “distance experience” (Dewey, 1930: p. 58) from the habitual “contact experience” of the macro-economic context of the country (Mische, 2009: p. 697), and vice versa. Analytically, positive or negative economic narratives might thus *counterbalance* the positive or negative role of the economic context on fertility intentions (HP2b).

### The Micro-Level Economic Situation

At the micro-level, the effects of narratives of the future on fertility intentions may be moderated by economic (labor-market) conditions. Unemployment is a crucial indicator of economic uncertainty and is frequently used in demographic research (e.g., Özcan et al., 2010; Schmitt, 2012). Negative theoretical effects of unemployment on fertility can be anticipated. On the one hand, unemployment erodes household financial resources by reducing the family income, which in turn often decreases or inhibits the desire for children (*income effect*). On the other hand, unemployment might facilitate the decision to have a (or another) child by providing additional time for childbearing and childrearing (*substitution effect*). More recently, with the rise of time-limited employment in Europe, there is a growing literature on the effects of these types of jobs on fertility (e.g., Pailhé & Solaz, 2012). Time-limited employment often reflects a low level of labor-market integration, which is connected to low employment protection and wage penalties, and may translate into feelings of economic uncertainty for individuals (Scherer, 2009; Schmitt, 2012; Vignoli, Tocchioni, et al., 2020). Our third research question is (RQ#3): *Do individuals’ labor-market conditions moderate the effect of the narratives of the future on fertility intentions*?

When facing negative economic and labor-market narratives of the future, both the jobless and those with jobs with uncertain conditions may have lower fertility intentions than employed individuals with permanent contracts because of the expected *twofold disadvantage* of the present and future condition. Indeed, the expectations produced by the negative narrative of the future may well amplify a critical situation characterized by a personal lack of income and employment instability (HP3a). However, following the socio-psychological uncertainty reduction framework (Friedman et al., 1994: p. 383), individuals—particularly women—may respond to unfavorable employment prospects by choosing to become parents. Hence, individuals already within economically disadvantaged settings may enhance their fertility intentions when exposed to a negative economic narrative of the future (HP3b). Alternatively, a positive narrative of the future may *counterbalance* unfavorable circumstances, producing a “distance experience” from the “contact experience” of daily routine (Dewey, 1930: p. 58; Mische, 2009: p. 697). Hence, those in unfavorable employment situations may value positive narratives of the future more so than others, fostering fertility intentions (HP3c).

When facing positive economic and labor-market narratives of the future, similar effects may be anticipated. Those with favorable employment prospects may seek confirmation of their security in positive narratives of the future, and their fertility intentions may thus be enhanced by this *twofold advantage* (HP3d). On the contrary, the negative narrative of the future may generate a “distance experience” from the daily routine for those with favorable employment conditions, which could result in a counterbalancing effect that depresses their fertility intentions (HP3e).

## A Laboratory Experiment

### Experiment Description

The causal effect of exposure to certain economic narratives of the future on fertility (intentions) is difficult to assess with observational data. Laboratory experimentation, however, allows one to explore this causal relationship. Laboratory experiments are typically conducted in a physical location selected by the researcher to maintain a high degree of control over treatments and other experimental conditions. Our laboratory experiments were organized at the University of Florence and at the University of Oslo^5^ between June 2019 and early February 2020—which is to say, prior to the COVID-19 pandemic. Our participants consisted of 837 couples (1674 participants in total) in which the women were aged between 20 and 40. We also ensured a balanced participation of jobless, permanently employed, and temporarily employed individuals. This allowed us to test the heterogeneous impacts of narratives of the future according to different pre-experimental settings (Italy vs Norway) and personal labor-market conditions.

The participants were recruited through the services of specialized survey agencies, without any anticipation about the content of the experiment (i.e., no references were made to family or economic aspects). They were asked to answer a large array of demographic, socio-economic, and psychological questions. The experiment was implemented using the O-TREE open-source platform (Chen et al., 2016). The experimental protocol is presented in “Appendix 1”.

**Fig. 2:**
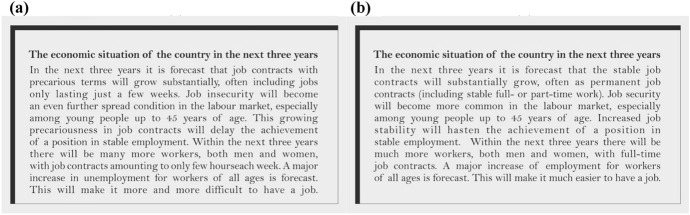
Mock newspaper article describing the future economic situation of the country; negative (**a**) and positive (**b**) scenarios

The narratives of the future were embodied by mock newspaper articles that were used as treatments. Each treated participant was asked to read (on a computer) a mock newspaper story describing a potential future economic scenario. We randomly assigned the participants to one of three groups. One group was exposed to a positive scenario (positive treatment), one to a negative scenario (negative treatment), and one was not exposed to any scenario (control group). The positive and negative scenarios focused on the same three economic aspects projected over the following three years: jobs with uncertain conditions (juxtaposition of permanent and temporary employment); instability of professional careers (whether the young will be able to secure a stable position or not); and joblessness (chances to gain or lose employment). The negative treatment consisted of reading a short news item describing a surge in precarious contracts, especially among the young, an increase in short-time jobs, and a rise in unemployment (Fig. 2a). The article in the positive treatment described a surge in permanent contracts, especially among the young, an increase in full-time jobs, and a rise in employability (Fig. 2b). The random treatment assignment allowed us to compare groups with similar characteristics, and thus make inferences about causation.

We next asked the participants to imagine themselves in the described future scenario and rate their fertility intention for the next three years (Q: “Do you intend to have a child in the next three years”). In order to ensure a pure priming effect, we asked no questions in between the treatment exposure and the surveying of intentions. Following recommendations from psychology literature, to grasp individual differences in psychological constructs with acceptable levels of precision (MacCallum et al., 2002), fertility intentions were assessed on a scale of 0 to 10, where 0 corresponded to “definitely not” and 10 to “definitely yes.” This choice allowed us to address both the direction and intensity of fertility intentions. The intermediate point of the scale was included so as also to capture ambivalent or neutral positions (Zammuner, 1998). Questions regarding intentions “in close temporal proximity to the prospective behavior” (Ajzen & Fishbein, 1973: p. 49) are generally considered suitable predictors of actual behavior (Philipov, 2009; Régnier-Loilier & Vignoli, 2011; Spéder & Kapitany, 2009).

### Sample and Statistical Analysis

Our sample was composed of 1,674 individuals (800 for Italy and 874 for Norway).^6^ 33.9% of the participants living in Italy and 34% of the participants living in Norway were placed in the control group (no scenario), 33.2% of the participants living in Italy and 30.9% of the participants living in Norway were exposed to the negative scenario, and 32.9% of the participants living in Italy and 35.1% of the participants living in Norway were exposed to the positive scenario. We analyzed the experimental data through means comparisons, given that randomization to treatment and control groups automatically controls for potential alternative explanations. Moreover, we performed a multivariable analysis by regressing fertility intentions via ordinary least square (OLS) regressions to include key socio-demographic control variables in the model equation. We employed cluster-robust standard errors at the couple level in models including both members of the couple.

While the scenarios (i.e., the mock newspaper articles) corresponded to the narratives of the future, we included a set of proxies for economic uncertainty related to past experiences and present condition in our analyses. These were: employment status and characteristics (1 = employed with a permanent contract; 2 = employed with a temporary contract; 3 = jobless); the level of equalized monthly household income (in EUR); and past experiences of joblessness (share of time spent jobless since the end of education).

We also included a set of variables to control for individual background: level of education, distinguishing between low (1 = elementary, junior high school, and short vocational courses), medium (2 = high school), and high education (3 = tertiary or higher); parity, dividing the childless (= 0) from parents (= 1); relationship status, distinguishing among individuals in a living apart together (LAT) relationship (= 1), married couples (= 2), and cohabiting couples (= 3); age, as a continuous variable; migration background (0 = natives; 1 = with a migration background); and the number of siblings (0 = no siblings; 1= one sibling; 2 = two siblings; 3 = three or more). We also included partner’s educational level and employment coded as for the participant.

Finally, we accounted for personality traits by incorporating a self-assessed measure of risk aversion to control how individuals feel, tolerate, and react to uncertainty. Specifically, we included the following question: “Would you describe yourself as someone who tries to avoid risk (risk averse) or rather as someone who is available to take a chance (risk taker)?” We asked our participants to rate their willingness to take risks on an 11-point scale, with 0 indicating complete unwillingness and 10 signifying complete willingness.

The analysis is organized as follows. To address RQ#1 and RQ#2, we evaluated the effect of the experimental condition on fertility intentions, stratifying our sample by gender and country. This allowed us to inspect RQ#1 (causal role of the narratives of the future on male and female fertility intentions) and RQ#2 (moderating role of different pre-experimental conditions in the two countries). For RQ#3 (the moderating role of personal employment states), we presented the findings by country and individuals’ employment conditions. We could not run an analysis by combining both labor-market conditions and gender due to the small sample size. Other variables*—*included in the model equation*—*could be theorized as moderators (i.e., age, parity, or education level). However, in preliminary analyses, their moderation effects did not emerge as relevant. Note that the experimental protocol imposed a balanced participation of individuals by different employment conditions so as to be able to test such a moderation effect and did not impose quotas for parity or education.^7^

The overall distribution of our analytical sample is reported in “Appendix 2”, Table 3.

## Results

Fertility intentions differed between participants exposed to future economic scenarios and those who were not (control group). We found a similar pattern for Italy and Norway, though with some differences in the magnitude of the effect. The mean answer to the 0–10 fertility intentions response scale for the control group was 4.9 in Italy and 4.5 in Norway. The mean answer for participants who read the negative scenario was lower: 4.2 in Italy and 3.3 in Norway. For those exposed to the positive scenario, the mean answer was higher: 6.9 in Italy and 5.2 in Norway. These initial findings provide evidence for a stronger effect of the positive scenario in Italy and a stronger effect of the negative scenario in Norway. We also performed a one-way analysis of variance (ANOVA), which highlighted a significant effect of the scenario on fertility intentions in both countries (Italy: *F* = 45.62, *p* < 0.001; Norway: *F* = 19.30, *p* < 0.001). Generally speaking, we found a clear impact of narratives of the future on fertility planning among the participants.

The experiment included a manipulation check to validate the quality of our treatment. We asked the participants to share their opinions on the description of the country’s economic situation from what they had read. Among those exposed to the negative scenario, 56.3% of the participants living in Italy and 64.2% of the participants living in Norway declared it to be highly negative, 30 and 26.9%, respectively, judged that it was mildly negative, and 12.6 and 6.3% considered it to be neither negative nor positive. Among those exposed to the positive scenario, 49.5% of the participants living in Italy and 56.5% of the participants living in Norway deemed it highly positive, 44.4 and 31.2%, respectively, assessed it to be only mildly positive, and 4.1 and 8.9% described it as neutral. Very few of the participants described the scenario unexpectedly. We conducted an ANOVA between subjects, and found there was a significant effect of the experimental condition on the manipulation check in both Italy and Norway (Italy: *F* = 13.95, *p* < 0.001; Norway: *F* = 5.89, *p* < 0.001).

**Fig. 3:**
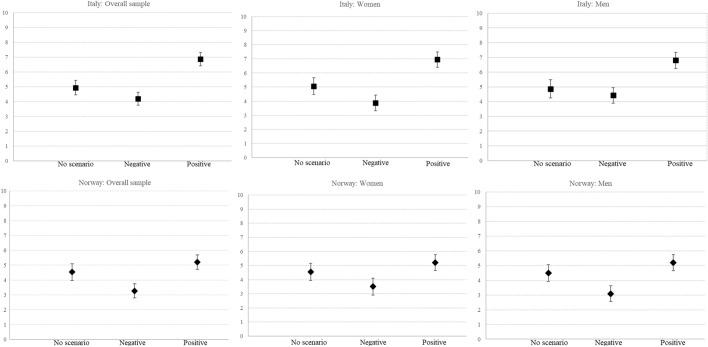
Predicted level of fertility intentions by treatment, gender, and country; with a 95% confidence band (*N*: Italy = 800; Norway = 874). For the pooled model, estimates from regression models include robust standard errors at the couple level. Models include all variables listed in Table 4 (“Appendix 2”)

A key advantage of an experiment of this kind is that randomization to experimental and control groups automatically controls for potential alternative explanations. We also performed a multivariable analysis by using an OLS regression that includes several key socio-demographic correlates of fertility intentions. Figure 3 shows the predicted level of childbearing intentions within the next three years by gender and country. The predicted values show the differences between the experimental and control groups, and between the positive and negative scenarios. The highest fertility intentions were found among participants exposed to the positive scenario (6.86 for Italy and 5.21 for Norway), while they were the lowest among the participants exposed to the negative scenario (4.19 for Italy and 3.27 for Norway). In Italy, women exposed to the positive scenario showed the highest predicted level of fertility intentions (6.95), while in Norway, the men exposed to the negative scenario showed the lowest predicted level of fertility intentions (3.09).

Regarding RQ#1, we found narratives of the future to have a clear impact in predicting fertility intentions. However, we did not find a clear gendered pattern. The positive scenario affected both men and women (HP1a), although we found this effect to be weaker in Norway. The negative scenario did not affect men in Italy (hence, HP1b is not fully confirmed), and we found no evidence of an uncertainty reduction framework among women (HP1d is not supported), thus confirming the negative effect (HP1c). These effects hold net of the markers of economic uncertainty related to past experiences and present condition: Table 1 reports the results of the model including the months of joblessness since the end of education, the current employment position, and the level of the couple’s income—i.e., the variables subsuming present status and past experiences. Notably, a couple’s higher income was associated with higher fertility intentions in both countries.

**Table 1:** Effects of randomized narratives of the future on fertility intentions, net of present status and past experiences

	Italy						Norway					
	Overall sample		Women		Men		Overall sample		Women		Men	
	Coeff	*p*-value	Coeff	*p*-value	Coeff	*p*-value	Coeff	*p*-value	Coeff	*p*-value	Coeff	*p*-value
Narratives of the future												
Scenario												
No scenario (ref.)												
Negative	− 0.750	0.025	− 1.182	0.005	− 0.448	0.279	− 1.283	0.000	− 1.942	0.019	− 1.428	0.000
Positive	1.913	0.000	1.90	0.000	1.925	0.000	0.723	0.052	0.734	0.084	0.722	0.074
Present status and past experiences												
Employment position												
Not working (ref.)												
Permanent job	0.587	0.090	0.699	0.141	0.927	0.093	0.117	0.739	0.838	0.079	− 0.964	0.067
Temporary job	0.508	0.163	0.024	0.797	1.254	0.029	0.363	0.326	0.712	0.168	− 0.364	0.527
Couples’ income	0.004	0.000	0.003	0.017	0.005	0.002	0.003	0.000	0.003	0.000	0.003	0.009
Months of unemployment	1.401	0.506	2.787	0.276	1.243	0.686	1.129	0.526	1.818	0.501	− 0.319	0.767

Results from OLS regression on fertility intentions (0–10) (*N*: Italy = 800; Norway = 874)

For the pooled model, estimates from regression models include cluster-robust standard errors at the couple level. Models include all variables listed in Table 4 (“Appendix 2”)

The interpretation of the effects of individuals’ background and personality traits (risk aversion) is beyond the scope of this paper (complete models are presented in Appendix, Table 4). Nevertheless, the effects of personal characteristics (e.g., age, parity, relationship status) all accorded with what has been found in previous literature for Italy (e.g., Rinesi et al., 2011) and Norway (Dommermuth et al., 2011), thereby indirectly validating our model. Individuals’ levels of risk aversion—a crucial trait in the study of decisions under uncertainty—did not affect fertility intentions in Norway. However, in Italy, higher levels of willingness to take risks corresponded to higher levels of fertility intentions, but only among women.

To answer RQ#2, we examined whether pre-treatment conditions—namely, the country context—moderated the impact of economic uncertainty on fertility intentions (see Fig. 3). Despite the significant effects of both positive and negative scenarios in the two countries, what seems to emerge is the important role of positive narratives of the future for Italy and of negative narratives for Norway. In Italy, which has a seemingly turbulent economic context, the positive economic narrative seemed the most relevant in affecting fertility intentions. In Norway, where the economic and financial context has been relatively stable in recent decades, the negative narrative of the future appeared particularly important. Notably, in Italy, the negative scenario had no statistically significant effect on fertility intentions—the same is true for the positive scenario in Norway. Therefore, we found evidence of a counterbalance effect (HP2b), while the hypothesis of the amplification of the context’s condition was unconfirmed (HP2a). The analyses illustrated no clear gendered pattern.

**Fig. 4:**
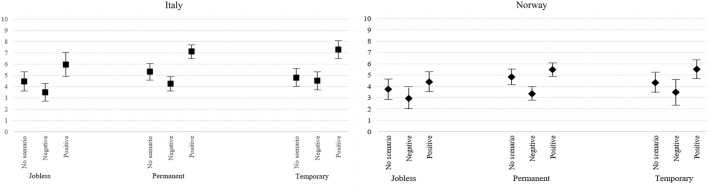
Predicted level of fertility intentions by treatment, country, and employment condition; with a 95% confidence band. Results from OLS regression on fertility intentions (0–10) (*N*: Italy = 800; Norway = 874). Notes: Estimates from regression models with robust standard errors at the couple level. Models include all variables listed in Table 4 (“Appendix 2”)

To answer RQ#3, we examined the role of narratives of the future in relation to diverse positions on the labor market. Figure 4 displays a clear pattern: higher fertility intentions for those exposed to the positive scenario, and lower fertility intentions for those exposed to the negative scenario. However, especially in Italy, and to a lesser extent in Norway, the effect of the positive scenario was stronger for those experiencing employment instability; especially for those employed in temporary positions. This result confirms our hypothesis of a *counterbalance* effect (HP3c). The effect of the negative scenario consistently produced statistically precise estimates only for those holding permanent positions—most notably for those living in Oslo—thereby again supporting the hypothesis of a *counterbalance* effect (HP3e). Overall, participants with current experience in higher employment instability were especially affected by a positive narrative of the future, and vice versa.

## Conclusions

The future is more and more often less predictable, as the COVID-19 disaster all too drastically proved. This study posits that the rise of uncertainty is key to understanding contemporary fertility dynamics. Uncertainty encompasses objective states, and recent research has highlighted the salience of factors such as a broader perception of uncertainty, which has been typically overlooked by traditional economic and labor-market indicators, and rose to prominence in the aftermath of the Great Recession (Comolli et al., 2021; Matysiak et al., 2021). In this article, we advance that explanations for fertility decisions should take into account the capacity to imagine the future, and argue that socially conveyed narratives of the future can influence the decision-making process. We tested this hypothesis by organizing an ad hoc laboratory experiment in Italy and Norway. We assessed fertility intentions after exposing individuals to a specific future economic scenario embodied in a mock newspaper article. Contrary to most fertility investigations in wealthy countries, our operationalization of uncertainty is thus forward-looking. The results highlight a clear causal impact of narratives of the future on fertility intentions among the participants to our lab experiments. The pattern of this influence is coherent in both contexts and among different social groups: the positive narrative of the future positive influences fertility, whereas the negative narrative has the opposite effect. We did not find a clear gender pattern in either of the two studied settings. Our findings are confirmed when the estimates are adjusted for several labor market-related variables commonly employed in the literature: the effects of the narratives of the future persist net of the past and contingent economic condition of the individuals.

Our results demonstrate the importance of the country-context of the participants. On the one hand, Italy persistently faces high levels of unemployment, especially among the young (Boeri & Jimeno, 2016; Tomić, 2018), and is a non-generous welfare state that lacks family policies. Norway, on the other hand, is one of the few European countries that emerged from the Great Recession relatively unscathed, and is characterized by its solid welfare state and reconciliation policies that favor fertility choices. In principle, the effects of the new economic narrative of the future may interact with such contexts in two ambivalent ways: by amplifying or counterbalancing the characteristics of the context. Only the hypothesis of a *counterbalancing effect* is supported by our results. Participants living in Italy reacted more strongly to the positive scenario than to the negative one, whereas the participants living in Norway were more influenced by the negative scenario. Nonetheless, several other unobserved patterns, such as culture or religion, may play important, or perhaps larger, roles in what causes people to believe in the scenario—the plausibility of the different scenarios may vary from country to country, thereby affecting their rates of being believed—and adapt their fertility intentions accordingly.

The impact of the narratives of the future also varied according to personal labor-market conditions, indicating that individual employment situations moderate the impact of these narratives. In particular, and coherently with the macro-level moderation effect, the causal effects of narratives of the future on fertility intentions proved especially powerful when the narratives differed most from the actual economic situation experienced by our participants. Namely, the positive economic scenario had the strongest positive impact on the fertility intentions of those with jobs with uncertain conditions. The effect of the negative scenario, on the other side, was strongest for those holding a permanent position. These results contrast with the *twofold dis/advantage* hypothesis: the negative/positive future scenario has a greater impact on the participants with an actual economic condition that differs most from that envisioned by the narrative of the future. Hence, the narrative of the future has a *counterbalancing effect*. Indeed, we observed (at the micro-level also) a greater effect of negative narratives for a more favorable economic condition and a greater effect of the positive narrative for a less favorable economic condition. The counterbalancing moderating role of micro-economic conditions thus diverts us from pursuing the hypothesis of a direct determinism of the uncertain objective status on fertility intentions.

In sum, the effects of objective economic situations on fertility intentions only account for part of the motivation driving fertility planning; narratives of the future also play a highly relevant role. Moreover, we observed the strongest reactions to each scenario when the envisioned situation diverged most from the current micro- or macro-economic situation. Indeed, narratives of the future create a *distance experience* from the ordinary life (Dewey, 1930; Mische, 2009), at both the macro- and micro-levels, that plays a potent role by inhibiting or facilitating fertility decision-making.

This study has several limitations. First, while our analytical strategy allowed us to offer causal evidence about the impact of narratives of the future on fertility intentions, this is inherently limited to the participants of our experiment. The external validity of our findings remains to be evaluated with larger, representative samples. Nonetheless, we believe that our experimental setting still affords valuable insights into the field for several reasons. The three groups were created with a random process that allowed us to include individuals with similar demographic characteristics in each. Furthermore, the participants were not university students—as it is frequently the case in economics laboratories—but individuals belonging to real couples of reproductive age. In any event, the unbalanced composition of our sample (e.g., by education) is a key reason why—further to the randomization approach—we performed regression analyses including control variables. Second, we are aware that the country-level moderation effect is likely due not only to the different economic contexts (i.e., varying levels of resilience to adverse economic shocks and different economic trends) but also to other factors (e.g., different welfare regimes or national cultures). Third, the limited sample size inhibited the stratification of the analysis by age, parity, or education, despite the fact that such distinctions have been shown to be important in previous research (e.g., Billari et al., 2009). Fourth, by considering only individuals in a partnership, we thus focused on a specific group. For instance, we excluded economically disadvantaged individuals who may struggle to find partners (Vignoli et al., 2016). This may further skew our estimates of the effect of narratives of the future on fertility intentions. Finally, we collected fertility intentions by using a 0–10 response scale, which limits the comparability with other studies employing a categorical fertility intention variable. Additionally, the surveying of fertility intentions employed in this study also prevents the possibility of examining “uncertainty” in the responses. Nevertheless, the complex nature of contemporary fertility decisions involves a much higher degree of ambivalence than in the past (Rotkirch, 2021), the large share of which changes across an individual’s life course (Kuhnt et al., 2020).

To the best of our knowledge, this study is the first experiment to establish a causal effect of narratives of the future on fertility intentions. While much of the literature has related fertility intentions or behaviors to (cumulative) past life events (e.g., unemployment or joblessness: Busetta et al., 2019; Pailhé & Solaz, 2012) or objective measures of uncertainty (e.g., unemployment or limited-time jobs: Barbieri et al., 2015; Hanappi et al., 2017; Kreyenfeld et al., 2012; Modena et al., 2013; Raymo & Shibata, 2017; Vignoli et al., 2012), we are here instead considering a pure forward-looking effect of economic uncertainty on fertility intentions. Our results suggest that a simple manipulation of future shared narratives affects fertility intentions. Due to the advances in communication technology, modern-day individuals are exposed to a continuous (over-)flow of information, which was further boosted after the outbreak of the current pandemic (Altig et al., 2020). This exposure is likely to increase individual feelings of uncertainty about the future because of the prevailing sensationalist or pessimistic tone and angles of media content. We propose that a focus on narratives of the future will help scholars gain a more comprehensive understanding of contemporary fertility patterns. With this paper, we thus hope to set the stage for future studies that seek to address the role of narratives of the future in fertility research. After all, it is not only the narratives of how the economy will develop which affects fertility choices. The role of “other narratives,” such as those regarding the possibility to combine paid work and family life of prospective parents, may be equally important and worth exploring in future research.

## Data Availability

The datasets generated during and/or analyzed during the current study are available from the corresponding author on reasonable request.

## Appendix 1 Experimental Protocol

The experiment was planned by a multidisciplinary team comprising demographers, sociologists, social psychologists, and economists. The team met several times throughout the course of one year to design the experiment and questionnaire. The experimental setting was implemented using the O-TREE open-source platform. A first pilot was organized in December 2017 with 200 students from the Department of Education, Languages, Interculture, Literature, and Psychology (FORLILPSI) at the University of Florence. The aim was to test the very first version of the text scenarios used in the experiments. After the pilot, the research group prepared a preliminary version of the full questionnaire. The first full-experiment pilot took place on September 8, 2018, at the University of Florence. A first scientific workshop was then organized on October 4–5 at the same university. Nine international experts from the aforementioned disciplines participated in the workshop and discussed the general experimental protocol and results of the pilot tests. After the workshop, we slightly modified the framing of the treatment and questionnaire to accommodate the feedback received. The protocol was approved by the Ethics Committee of the University of Florence and was then updated after the first full-experiment pilot and first scientific workshop, whereupon it was once again approved by the Ethics Committee.

The laboratory experiments were conducted at the University of Florence and at the University of Oslo. The laboratory experiments in Italy were carried out during five sessions between May 2019 and February 8, 2020. Those in Oslo began in September 2019 and in November of the same year; they required 16 sessions because of the reduced capacity of the rooms. Two agencies, one in Florence and one in Oslo, were selected to recruit the couples in which the women were aged between 20 and 40. A balanced participation of jobless, permanently employed, and temporarily employed individuals was required. The agencies were also asked to try to ensure a distribution by education and parity that would mimic that of the national population distribution, but without imposing strict quotas. Participants were selected from the agencies’ available panel participants, boosted with additional participants recruited through advertisements distributed in public places, without showing any information about the experiment’s content. The participants were paid 50 euros per couple in Italy and 40 kroner per couple in Norway. They could add a further 0–50 euros and 0–40 kroner per couple by answering a lottery question. Incentivized lotteries are a standard way of measuring risk attitude in the economic domain and they require the payment of one of the decisions taken by the subject, randomly chosen (see descriptions of Section E and F—though these incentivized questions are not used in the present paper).

The structure of the experiment is described in Table 2. The treatment consisted in the reading of a short mock newspaper article. After reading the text, the participants were immediately presented with a series of items/scales with which to measure their fertility intentions (see the experiment description section for details about sections A and B). Once done, we asked a series of questions (section C) based on the theory of planned behavior (Ajzen, 1991) applied to fertility intentions (Ajzen & Koblas, 2013). We then collected information regarding individuals’ intentions, attitudes, subjective norms, and perceived behavioral control.

**Table 2:** Protocol structure

	No scenario	Scenario	Mean time (in sec.)
A. Scenario		✓	47.7
B. Questions about fertility intentions	✓	✓	49.2
C. Questions based on the Theory of Planned Behavior	✓	✓	322.7
D. Open questions about uncertainty	✓		Time not recorded
E. Lotteries/batteries to measure risk aversion, loss aversion, and time preferences	✓	✓	513.0
F. Raven matrices test (selection) and logic test	✓	✓	633.0
G. Socio-demographic information	✓	✓	690.4

The members of the couples in the control group were not given a scenario to read, and their experiment instead began with questions about fertility intentions and the psychosocial items. They were then requested to answer open questions about the role of uncertainty in their lives (section C).

Sections E and F of the experimental session were devoted to assessing the individual traits of all subjects. We examined several dimensions of individual heterogeneity, including risk aversion, loss aversion, and time preferences (discount rates) (section E), as well as cognitive skills (section F). Person-specific time preferences were measured through a Kirby delay discounting questionnaire (Kirby et al., 1999). We assessed risk and loss aversion via two incentivized lotteries (Holt & Laury, 2002), one with a 50/50 gamble and one with unknown probabilities. The participants were paid both for their participation and according to their final lotteries’ payoffs. The fact that the participants’ remuneration depended on how much they won helped bolster the credibility of the behavioral results of the lottery. To have a proxy of cognitive skills, we used a set of Raven Matrices (Raven, 2000) and a Cognitive Reflection Test (Primi et al., 2016).

The final part of the survey was devoted to demographic questions about participants’ socio-economic conditions and habits (section G).

The approximate duration of the experiment and the survey was one hour. Table 2 specifies the mean time the participants devoted to each section. For this paper, we used answers from sections A, B, E, and G.

## Appendix 2

See Tables 3 and 4.

**Table 3:** List of variables by country and scenario

	Italy				Norway			
	Total	No scenario	Negative	Positive	Total	No scenario	Negative	Positive
Narratives of the future								
Scenario								
No scenario	33.9				34.0			
Negative	33.2				30.9			
Positive	32.9				35.1			
Present status and past experiences								
Employment condition								
Jobless	25.8	26.1	27.8	23.5	20.4	22.1	17.1	21.1
Permanent job	45.0	45.3	43	47	60.2	54.2	65.6	61.4
Temporary job	29.1	28.6	29.2	29.5	19.4	23.7	16.6	17.5
Couple income (mean)	2,209.56	2,140.60	2,132.51	2,358.19	4,528.54	4,506.17	4,580.69	4,504.38
Share of time in unemployment (mean)	0.031	0.031	0.035	0.026	0.030	0.028	0.026	0. .036
Individual background and traits								
Level of education								
Low	16.6	17.4	16.0	16.5	10.8	10.0	8.5	13.6
Medium	46.9	45.4	47.2	47.9	19.2	19.7	14.4	22.7
High	36.5	37.1	36.8	35.6	70.0	70.2	77.0	63.6
With child/children	53.7	52.5	54.1	54.7	65.5	63.1	65.6	67.9
Without children	46.3	47.5	45.9	45.3	34.5	36.9	34.4	32.1
Relationship status								
LAT (living apart together)	24.1	25.7	19.2	27.4	11.3	11.8	11.5	10.7
Marriage/civil union	32.6	31.2	37.8	28.8	29.2	31.5	27.0	28.9
Cohabitation	43.3	43.1	43.0	43.8	59.5	56.7	61.5	60.4
Age	33.6	33.8	33.9	33.0	30.4	30.4	30.6	30.1
Migration background	3.8	3.2	3.7	4.5	10.0	7.0	12.2	11.0
Sterility	2.3	1.8	1.8	3.4	2.2	1.3	2.9	2.3
Number of siblings								
0	19.9	18.8	20.4	20.5	7.4	8.0	7.8	6.5
1	52.8	55.1	50.0	53.8	36.1	38.8	35.1	34.4
2	16.6	17.0	18.1	14.5	31.1	28.1	33.5	31.8
3 or more	10.7	9.1	11.5	11.2	25.4	25.1	23.6	27.3
Risk aversion	5.9	5.7	6.0	6.0	5.0	4.8	5.1	5.1
N	800	272	264	264	874	298	268	308

**Table 4:** Effects of randomized narrative of the future on fertility intentions

	Italy						Norway					
	Overall sample		Women		Men		Overall sample		Women		Men	
	Coeff	*p*–v	Coeff	*p*–v	Coeff	*p*–v	Coeff	*p*–v	Coeff	*p*–v	Coeff	*p*–v
Narratives of the future												
Scenario												
No scenario (ref.)												
Negative	− 0.750	0.025	− 1.182	0.005	− 0.448	0.279	− 1.283	0.000	− 1.942	0.019	− 1.428	0.000
Positive	1.916	0.000	1.900	0.000	1.925	0.000	0.723	0.052	0.734	0.084	0.722	0.074
Present status and past experiences												
Employment position												
Not working (ref.)												
Permanent job	0.587	0.090	0.699	0.141	0.927	0.093	0.117	0.739	0.838	0.079	− 0.964	0.067
Temporary job	0.508	0.163	0.024	0.797	1.254	0.029	0.363	0.326	0.712	0.168	− 0.364	0.520
Couple's income	0.004	0.000	0.003	0.047	0.005	0.002	0.003	0.000	0.003	0.000	0.003	0.009
Months of unemployment	1.401	0.506	2.787	0.276	1.243	0.686	1.129	0.526	1.818	0.501	− 0.319	0.767
Individual background and traits												
Gender (ref. men)	− 0.154	0.435					0.121	0.477				
Education												
Low (ref.)												
Medium	0.189	0.573	− 0.307	0.578	0.448	0.358	− 0.398	0.374	− 0.990	0.191	− 0.064	0.872
High	0.335	0.396	0.583	0.372	0.095	0.577	0.625	0.112	0.350	0.604	0.845	0.125
Number of children												
No children (ref.)												
With child/children	− 1.137	0.000	− 0.773	0.067	− 1.198	0.004	− 0.679	0.126	− 0.326	0.544	− 0.946	0.046
Relationship status												
LAT (Living Apart Together) (ref.)												
Marriage/Civil Union	0.962	0.023	0.989	0.060	0.962	0.060	1.287	0.013	1.040	0.126	1.615	0.006
Cohabitation	0.7439	0.027	0.835	0.052	0.630	0.140	1.444	0.000	1.442	0.009	1.576	0.000
Age	− 0.083	0.002	− 0.121	0.006	− 0.077	0.017	0.006	0.848	− 0.034	0.453	0.010	0.683
Country of origin												
Immigrants	− 0.396	0.548	0.337	0.502	− 0.875	0.410	0.165	0.679	0.492	0.375	− 0.656	0.283
Sterility	− 0.747	0.351	− 2.038	0.218	− 0.026	0.852	− 1.151	0.243	− 1.498	0.114	− 0.334	0.824
Number of siblings												
0 (ref.)												
1	0.082	0.786	0.370	0.418	− 0.214	0.623	− 0.388	0.442	− 0.534	0.484	− 0.036	0.877
2	− 0.121	0.761	0.120	0.835	− 0.509	0.360	− 0.098	0.534	0.129	0.867	− 0.151	0.651
3 or more	− 0.382	0.441	− 0.957	0.191	0.066	0.787	0.172	0.492	0.398	0.878	0.508	0.451
Partner’s education												
Low (ref.)												
Medium	0.612	0,058	0.018	0.760	1.376	0.015	− 0.332	0.485	0.113	0.870	− 0.895	0.210
High	0.660	0.090	0.025	0.786	1.597	0.010	0.265	0.526	0.515	0.416	− 0.226	0.722
Partner’s employment												
Not working (ref.)												
Permanent job	− 0.063	0.857	0.319	0.446	− 0.484	0.310	0.232	0.481	− 0.554	0.291	0.858	0.043
Temporary job	− 0.252	0.487	0.693	0.204	− 0.992	0.030	0.333	0.363	− 0.242	0.467	0.839	0.089
Risk aversion	0.163	0.016	0.159	0.025	0.156	0.078	0.072	0.654	0.082	0.567	0.063	0.547
R^2^	0.188		0.221		0.215		0.144		0.160		0.160	

Results from OLS regression on fertility intentions (0–10) (*N*: Italy = 800; Norway = 874)

For the pooled model, estimates from regression models include cluster-robust standard errors at the couple level

## Footnotes

1 The salience of uncertainty within the decision-making process has been recently re-discovered thanks to the work of Jens Beckert. The first part of Beckert’s 2016 book is an action theory under uncertain conditions that can be applied to different life domains. For the remainder of that book, Beckert applies this theory to different aspects of capitalism, though we should note that this is not the only possible application. Beckert’s action theory comes from the pragmatist tradition of Mead and Dewey—and its recent developments by Hans Joas—that was not entirely focused on economic dynamics. Their tradition, indeed, provided the concepts and analytical strategies with which to understand how people cope with uncertainty. The experience of uncertainty over the future is part of long-term plans, such as fertility decisions.

2 The use of the concept of narratives is widespread across the social sciences. While the analysis of narratives is a traditional research method in anthropology and sociology, the interest in narratives was popularized within psychological research thanks to the seminal works of Sarbin (1986) and Bruner (1986). Moreover, the study of narratives was recently promoted in economics with the Shiller’s “Narrative economics” (2020). However, while the economic study of narratives focuses on the capacity of viral stories to influence economic decisions, our use of the concept relies on the pragmatist tradition of Mead (1932) and Dewey (1930) that views narratives as a necessary tool to cope with uncertainty. “Narratives laid out in forecasts transform uncertainty into a fictitious certainty contributing to decision-making […] Convincing narratives of the future […] are the tools actors need to make decisions that would otherwise seem random” (Beckert 2016: p. 242). Narratives have the capacity to create an actionable path in uncertain conditions, as in the case of long-term plans (Mische 2009; Tavory & Eliasoph, 2013). Fertility plans, indeed, entail the capacity to re-configure a single narrative from the undecided scenario of ordinary routine (Vignoli et al., 2020a).

3 The Norwegian survey used in Dommemuth et al. (2005) does not distinguish those who *definitely* intend to have a child from those who *probably* intend to have a child.

4 Despite our specific focus on economic patterns, we recognize the various differences between the two countries. Most notably, it is well-known that Norway is a more secularized society than Italy (Lesthaeghe, 2020).

5 Although we refer to countries when presenting our results, we acknowledge that the responses are not representative of Italy or Norway.

6 A power analysis using G*Power (Erdfelder et al., 1996) indicated that a minimum sample of 252 would be needed to detect medium effects (effect size = 0.25) with 95% power using an ANOVA with the alpha at 0.05.

7 Not imposing quotas by education results in unbalanced samples. For instance, more highly educated individuals are overrepresented in both countries—i.e., our sample includes 70% of highly educated participants living in Norway in contrast to 36.5% living in Italy. As such, in addition to the randomization, we also estimated multivariable models by controlling for education level.
